# Performance Assessment of All-Solid-Waste High-Strength Concrete Prepared from Waste Rock Aggregates

**DOI:** 10.3390/ma18030624

**Published:** 2025-01-29

**Authors:** Yunyun Li, Meixiang Huang, Jiajie Li, Siqi Zhang, Guodong Yang, Xinying Chen, Huihui Du, Wen Ni, Xiaoqian Song, Michael Hitch

**Affiliations:** 1 Key Laboratory of Resource-Oriented Treatment of Industrial Pollutants, School of Resource and Safety Engineering, University of Science and Technology Beijing, Beijing 100083, China; lyyustb@163.com (Y.L.); 13657814664@163.com (M.H.); zsq2017@ustb.edu.cn (S.Z.); 17866710223@163.com (G.Y.); 18401619832@163.com (X.C.); niwen@ces.ustb.edu.cn (W.N.); 2 China Institute of Urban Governance, Shanghai Jiao Tong University, Shanghai 200030, China; songxq@sjtu.edu.cn; 3 Faculty of Science, University of the Fraser Valley, Abbotsford, BC V2S 7M8, Canada; michael.hitch@ufv.ca

**Keywords:** waste rock, washed aggregate, mechanical properties, durability, porosity

## Abstract

In order to solve the problems of the large-scale resource utilization of iron ore waste rock, waste rock is used to prepare green building materials, but it needs to be further promoted for use in high-strength concrete. In this study, high-strength concrete was prepared using iron ore waste rock as coarse and fine aggregates combined with solid waste-based cementitious materials. The mechanical and durability properties of washed and unwashed concrete with two types of aggregates were compared, including compressive strength, freeze resistance, chloride ion resistance, carbonation resistance, pore distribution, microstructural characteristics, and environmental and economic benefits. The results indicated that water-washing pretreatment significantly reduced the stone powder content of waste stone aggregate from 14.6% to 4.5%, which had a significant effect on the basic properties of concrete. The compressive strength of concrete with water-washed waste rock aggregate was 61 MPa, 64.9 MPa, and 68.8 MPa at 28, 56, and 360 days, respectively, with long-term stability. The washed aggregate concrete had a porosity of less than 4%, freeze-resistant grade of F200, 28 d electrical flux <500 C, and a carbonation depth of less than 10 mm. The improved performance of the washed aggregate concrete was attributed to the fact that after washing pretreatment, the water absorption of the aggregate was reduced, the cementitious materials were fully hydrated, and the internal microstructure was denser. The high-strength concrete prepared in this study effectively used iron ore waste rock and solid waste-based cementitious materials, which not only reduces environmental burden but also provides basic data references for future engineering applications using iron ore waste rock aggregate concrete.

## 1. Introduction

The mining industry generates a large amount of solid waste every year, amounting to about 20,000 to 25 billion tons [1]. The huge accumulation of mine tailings and waste rock has not only occupied land areas but has also led to high management and maintenance costs, which have had a negative impact on the environment and ecology. The resource utilization of tailings waste rock has become a global problem. Concrete, as a widely used building material, consumes a large amount of sand, stone, cementitious materials, etc., every year, and is thus an important field for the extensive resource utilization of tailings waste rock [2]. With 15% of the country’s total iron ore reserves, China’s Anshan region has the largest concentration of iron ore in the country [3,4]. Waste rock refers to the large amount of non-mineralized surrounding rock and entrained rock produced during the extraction of mineral resources [2]. Mine tailings are industrial solid wastes generated after ore beneficiation [5]. Mining operations generate approximately 65 to 80 billion tons of waste annually, of which 10 to 15 billion tons are tailings, with the remainder being waste rock [6,7]. Currently, the utilization of iron ore tailings waste rock includes filling materials, engineering applications, iron resource recovery, aggregates, etc. [8,9,10]. Compared with tailings, the utilization of waste rock has received less attention. The annual consumption of concrete in China has reached 7 billion t [11]. With the rapid development of China, the amounts and demand for high-strength concrete are gradually expanding. Among them, aggregate accounts for three-quarters of the volume of concrete, with a huge consumption of more than 349 million tons of aggregate per year [12,13], and natural river sand is in short supply. As a result, the use of waste rock as aggregate can not only solve the problem of the shortage of natural aggregates but also massively dissipate the stockpiled waste rock and relieve the harm brought to the ecological environment.

It has been a long-standing problem that natural sand is scarce in some industrialized countries and regions. Manufactured sand to replace the natural sand preparation of concrete has become an inevitable trend for the sustainable development of the concrete industry. Manufactured sand is waste rock crushed by mechanical equipment; although, after the screening process, there is still a small amount of stone powder residual with fine particle sizes of less than 0.75 mm [14]. Stone powder is an essential factor affecting the quality of manufactured sand. Stone powder content that is too high harms the strength development and durability of concrete, especially in the preparation of high-strength concrete. Despite the fact that manufactured sand is now widely used in mortar and concrete, it is worth noting that some generally accepted views usually regard it as a poor-quality sand that is mostly used in low- and medium-strength concrete [15]. When the stone powder content is within 10%, it can play a certain role in filling in low- and medium-strength concrete and has no significant negative impact on durability [16,17]. A wide range of studies have reported a stone powder content of 10–20% as a threshold for the preparation of low-strength concrete with manufactured sands [1,18,19]. In recent years, a lot of work has been carried out by a number of researchers on the effect of stone powder on the properties of high-strength concrete. Ma et al. [20] used a water-washing pretreatment for manufactured sand of granite, tuff, and limestone lithologies to prepare ultra-high-performance concretes with a 28-day compressive strength of more than 150 MPa. The study focused on granite mechanism sand and limestone mechanism sand and reported that the binder was still dominated by cement [21,22]. In 2020, when world cement production reached 4.1 billion tons, global emissions from CO_2_ cement production totaled about 2.0 Gt [21]. Therefore, in order to reduce CO_2_ emissions, major actions have been aimed at replacing or reducing the clinker content of Portland cement, including as a solid waste-based cementitious material. Iron tailings waste rock can be successfully used in an increasing number of conventional concretes, but still needs to be promoted for use in high-strength concretes. There are more difficulties in preparing manufactured sand from magnetite quartzite type waste rock, in which the content of stone powder limits the use of this type of mechanism sand [23]. Although researchers have recognized the importance of pretreatments such as that of washed sand in the production of manufactured sand, the existing literature mainly focuses on concrete proportion and workability, and there is still a lack of data support and in-depth discussion for the preparation of high-strength concrete from quartzite iron tailings waste rock and solid waste-based cementitious materials.

**Figure 1:**
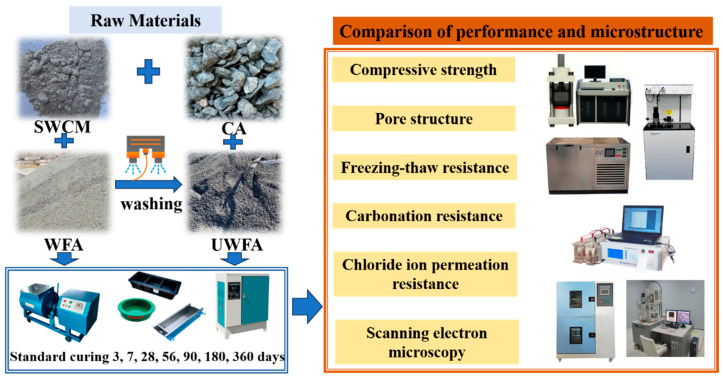
Experimental design. Note: SWCM: Solid waste cementitious materials. CA: Coarse aggregate. WFA: Washed fine aggregate. UWFA: Unwashed fine aggregate.

The objective of this study was to evaluate the mechanical and durability properties of high-strength, all-solid-waste concrete estimated from waste stone aggregates by reducing the stone powder content of waste stone aggregates in combination with solid-waste based cementitious materials using quartzite-type iron tailings waste stone as the object of study (Figure 1). Reducing the stone powder content and exploring the possibility of preparing high-strength concrete by combining solid waste-based cementitious materials with waste stone aggregates can be recommended for practical construction activities and has guiding significance for the future application of waste stone manufactured sand on a large scale.

## 2. Materials and Experimental Techniques

### 2.1. Materials

Solid waste-based cementitious materials: The cementitious materials were made from 60% S95 slag and 40% semi-dry desulfurization ash after mixing and grinding for 40 min. The composition of the cementitious materials is shown in Table 1 and the performance indexes are shown in Table 2.

Aggregate: Waste rock aggregate, i.e., waste rock fine aggregate (0~10 mm) and waste rock coarse aggregate (5~25 mm), was produced by crushing a waste rock pile of the Dagushan iron ore mine. In the test, the aggregate was washed with water through a square hole sieve of 1.25 mm and 80 μm. After removing the material below 80 μm and controlling the moisture outside, the chemical composition content of the aggregate and the contents of stone powder and mud lumps were detected. The chemical composition of washed and unwashed aggregates is shown in Table 3 and the content of mud and stone powder is shown in Table 4.

The stone powder content of the untreated fine aggregate was 14.6%, which exceeded the requirement of 10% stone powder content for fine aggregate, as stipulated in JGJ52. The density of fine aggregate was 1570 kg/m^3^, the fineness modulus was 3.3, the porosity was 42%, and the crushing index was 15.8%. The waste rock coarse aggregate density was 1480 kg/m^3^, the porosity was 45%, and the crushing index was 7.9%.

**Figure 2:**
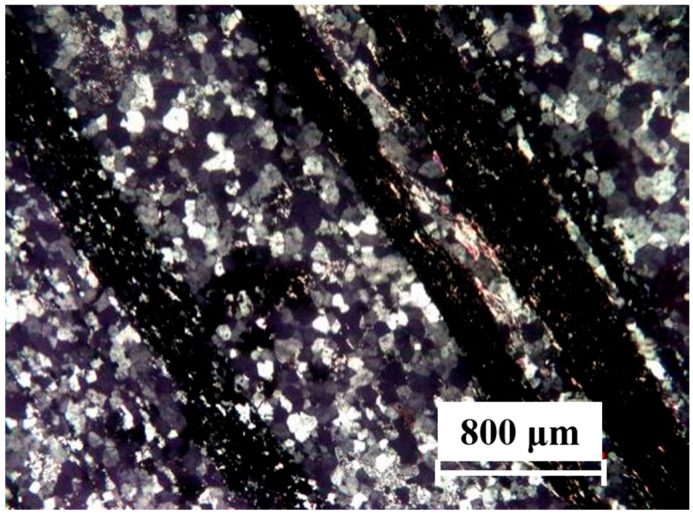
Microscopic characterization of the waste rock lithology (orthogonally polarized light).

The lithofacies of the Dagusan waste rock were identified and microscopically characterized, as shown in Figure 2. The identified lithofacies mainly consisted of quartz and sericite, magnetite, apatite, zircon, and opaque minerals. Among the identified minerals, quartz was in the form of it-shaped grains, 0.1–0.5 mm in size, mosaic-like, and with directional distribution as the central part of the rock; sericite was flaky, 0.001–0.05 mm in diameter, scattered, and with directional distribution; and a small portion of the aggregates were irregularly distributed in the form of piles. The rock was lightly cracked and there were opaque minerals along the fissures.

High-efficiency polycarboxylic acid water-reducing agent (water reduction rate 40%) from Shenyang Shengxinyuan Building Material Technology Co. (Liaoning, China) was used.

### 2.2. Mixture Proportion and Specimen Preparation

The experimental mixing proportions are shown in Table 5. The specimen was prepared according to the national standard GB/T 50081-2019. There were three specimen sizes in this experiment, which were 100 mm × 100 mm × 100 mm cube, Φ100 mm × 50 mm cylinder, and 100 mm × 100 mm × 400 mm prism. Cubic samples were used for strength tests, carbonization experiments, and other microscopic tests; cylindrical samples were used for electrical flux experiments; and prismatic samples were used for frost-resistance tests. The specimens were demolded after one day of molding and placed in a standard curing chamber to cure to a specific age.

### 2.3. Test Methods and Characterization Techniques

#### 2.3.1. Compressive Strength Test

According to the national standard GB/T 50081-2019, the compressive strengths of the different concrete specimens were tested at the corresponding ages of 3, 7, 28, 56, 180, and 360 days. Three specimens were tested in each group and the test results were averaged.

#### 2.3.2. Rapid Chloride Migration Test

Chlorine ion permeability was investigated using electric flux in accordance with the test method specified in GB/T50082-2009. After 28 days of standard curing, cylindrical specimens were sealed on the sides with epoxy resin and placed in a vacuum saturator for saturation and electric flux tests.

The anode solution was 0.3 N NaOH and the cathode solution was 3% NaCl. The tests lasted for 6 h and were recorded every 30 min. Three parallel specimens were set up in each group for the flux test and the results were calculated using the arithmetic mean method.

#### 2.3.3. Freeze–Thaw (F-T) Cycle Test

The freeze resistance of concrete was tested according to the rapid freezing method over a temperature range of −20 °C to 20 °C. Mass loss and relative dynamic modulus of elasticity were used to evaluate the frost resistance of the concrete, which was tested every 25 F-T cycles. The steps were as follows: (1) Specimens cured for 28 or 56 days were immersed in water at 20 ± 2 °C for 6 days. (2) Each specimen was numbered and its initial mass, M_0_, was measured. (3) Each F-T cycle was 3.75 h. The thawing time was more significant than 1/4 of the freeze–thaw time, with the freezing temperature at −19 °C, the thawing temperature at 6 °C, and the thawing time at 1.25 h. The freezing time was 2.5 h. (4) Each of the 25 F-T cycles was 3.75 h, with the thawing time greater than 1/4 of the freeze–thaw time. (5) The specimens were removed every 25 F-T cycles, the dynamic modulus of elasticity and mass *M_n_* were determined, and the specimens were returned to the F-T cycle machine to continue the experiment. The formula for calculating the rate of concrete mass loss was as follows:

(1) $$\Delta M_{n}=\frac{M_{n}-M_{o}}{M_{o}}\times 100\%$$

where Δ*M_n_* = mass loss rate (%), *M_n_* = mass of specimen after n F-T cycles (g), and *M_o_* = mass of specimen before F-T cycles (g).

When the relative modulus of elasticity of the specimen decreased to 60% or the mass loss reached 5%, the test was stopped.

#### 2.3.4. Carbonation of Concrete

Specimens for the carbonization test (100 mm cube specimens on day 28) were removed from the standard curing chamber 2 days before the test and dried in a desiccator (48 h at 40 °C). After drying and cooling, all surfaces of the specimens were sealed with heated paraffin. The treated specimens were placed on a shelf inside the carbonization chamber, with the exposed side surfaces facing up. Throughout the test, the relative humidity inside the chamber was controlled at 70 ± 5%, the temperature was controlled at 20 ± 2 °C, and the carbon dioxide concentration was maintained at 20.0 ± 0.0 °C. At 28 days of carbonization, the specimens were removed, and the depth of carbonization was determined by fracturing the specimens. Alcohol solution was used (1 g of phenolphthalein dissolved in 80 mL of ethanol and 20 mL of deionized water). After about 30 s, the depth of carbonization at each measurement point was measured with the phenolphthalein alcohol solution. The depth of carbonation was measured at each measurement point with a steel ruler. At least 5 points were measured on each carbonized surface, recording the arithmetic means of the carbonation depths.

#### 2.3.5. Pore Structure of Concrete

Mercury in Piezo (MIP) test samples were about 1 cm^3^ in size, with 5–6 small particles in each group. The test apparatus was a fully automated mercury piezometer (Auto Pore V 9620, Micromeritics, Norcross, GA, USA) with a maximum test pressure of 413 MPa [24].

#### 2.3.6. Microstructure Test

The microstructural evolution of hydrated concrete products was performed using the scanning electron microscopy (SEM) technique. The electron microscope was a SUPRA 55 manufactured by Carl Zeiss, Oberkochen, Germany. The analysis was conducted at an operating voltage of 10 kV and the vacuum was kept below 9.9 × 10^6^ mbar. The chemical composition of the samples was further determined by an energy-dispersive X-ray spectrometer (EDX), also provided by Carl Zeiss.

The microscopic lithology of waste rock was carried out using an BX53M series microscope (Olympus, Tokyo, Japan) with a combined reflective and transmitted light frame equipped with a 1.25–100× IR objective lens and an illuminator.

## 3. Results

### 3.1. Compressive Strength

**Figure 3:**
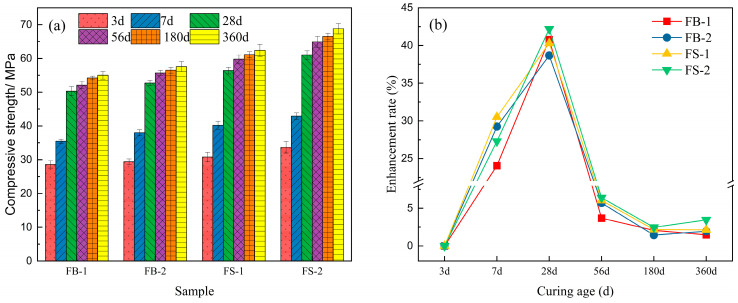
Compressive strength (**a**) and compressive strength enhancement rate (**b**).

Figure 3 shows the effect of the compressive strength of the concrete prepared with washed and unwashed aggregates. As can be seen from Figure 3, at different curing ages, the compressive strength of concrete prepared with washed and unwashed aggregates was not affected in the same way. When the curing age was 28 d, only FS-2 concrete specimens could reach 61 MPa, and the remaining three concrete groups were in the range f 50–56 MPa. The high content of stone powder in FB series aggregate could play a specific filling role in the early hydration stage, manifested in the small gap between the compressive strength of 3 d and 7 d compared with that of the washed FS series. However, the compressive strength of the FB series was significantly lower than that of the FS series after 28 d of the critical maintenance period. For instance, the compressive strength of FB-1 was 12.62% lower than that of FS-1 and that of FB-2 was 16.52% lower than that of FS-2 at 56 days of curing age. This was attributed to the fact that the source of concrete strength mainly relied on the hydration products to cement with the components to form a close-packed structure when the aggregate stone dust content was high, hindering the cementitious materials’ hydration reaction [25]. The compressive strength growth rate of both the FB and FS groups showed an increasing and then decreasing trend with the increase in curing age (Figure 3b). The maximum growth rate of compressive strength at 28 days was about 40%. The specimens of FS-2 cured for 360 days showed a growth of compressive strength of 3.46% to 68.8 MPa. This indicated that reducing the stone powder content of the waste rock aggregate helped to improve the stability of the mechanical strength of the concrete. This was mainly due to the fact that the aggregate washed away a large amount of stone powder and mud lumps, which reduced the water absorption rate of the aggregate and improved the sustained water molecules for later hydration [26]. C60 high-strength, all-solid-waste concrete could be prepared by simply washing the waste rock aggregate with water, and the strength of the concrete could be kept stable.

### 3.2. Pore Structure

**Figure 4:**
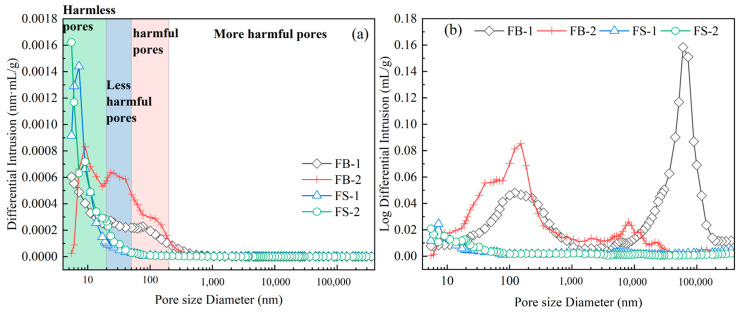
The pore structure of all-solid-waste concrete at age 56 d: (**a**) pore distribution, (**b**) total pore volume.

To further reveal the effect of stone powder on the pore structure in concrete with washed and unwashed aggregates, the pore structures of the hardened body specimens of FB series and FS series concrete were determined by using the mercuric pressure method, and the results are shown in Figure 4. The pores in the hardened body were categorized into harmless pores of 0~20 nm, less harmful pores of 20~50 nm, harmful pores of 50~200 nm, and harmful large pores of >200 nm, according to the pore meridian and the state of water presence in the hardened body [27]. As can be seen from Figure 4, the pores of the FB series were mainly divided into less harmful pores and harmful pores, while the FS series had mainly harmless pores.

**Figure 5:**
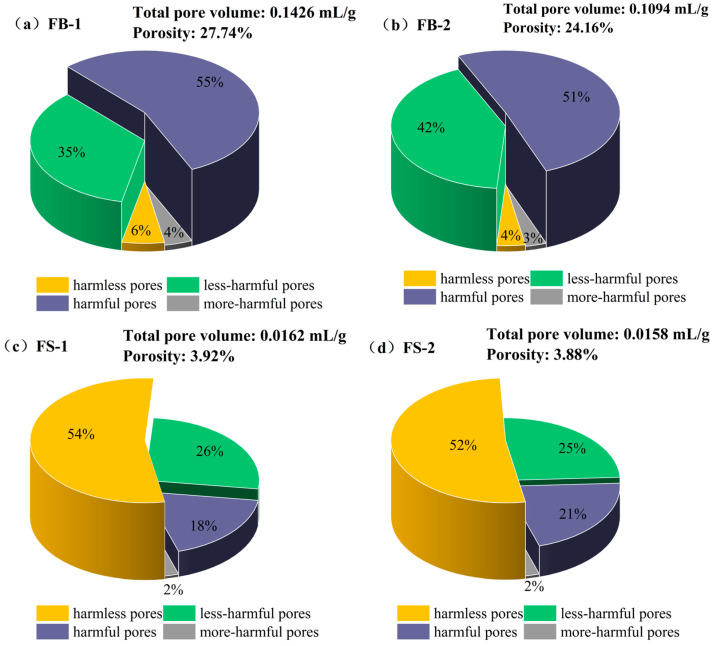
Pore size distribution of 56 d specimens of all-solid-waste concrete.

As can be seen from the pore size distribution of concrete in Figure 5, the porosity of the FS series concrete was significantly lower, the content of harmless and less harmful pores was higher, and the content of deleterious macropores was drastically lower than that of the FB series concrete. The porosity of FS-1 was 23.82% lower than that of FB-1 and the porosity of FS-2 was 20.28% lower than that of FB-1. This further indicated that the aggregate stone dust and mud clod content dramatically influenced the concrete structure. More than 50% of pores in the FB series concrete were harmful pores and only about 5% were harmless pores. After washing the waste rock aggregate, the harmful pores were reduced to about 20%, the less harmful pores were reduced to about 25%, and the harmless pores were increased to more than 52%. The reason can be summarized in two aspects [28,29]: firstly, it was the high content of stone powder and mud lumps in the aggregate that affected the hydration of the cementitious materials and the generation of C-S-H gel and AFt was lower, resulting in the reduction in harmless pores; secondly, the lower content of stone powder and mud lumps in the water-washed aggregate reduced the water absorption rate of the aggregate, and the cementitious materials could be sufficiently hydrated, which improved the system hydration in the middle and later stages of C-S-H gel generation, the polymerization degree of the C-S-H gel, and the degree of densification of the hardened body.

### 3.3. Durability Properties

#### 3.3.1. Freeze–Thaw Resistance

**Figure 6:**
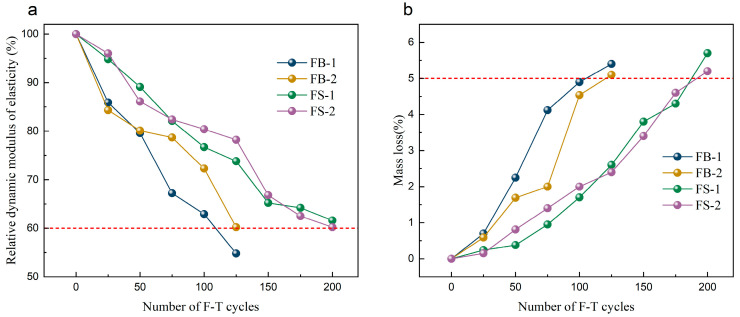
Concrete freeze–thaw cycle analysis: (**a**) mass loss rate, (**b**) relative dynamic modulus of elasticity.

The test materials were taken from northeast China, a cold region, so the prepared concrete needed to have good freeze-resistant properties. Figure 6 shows the test results of the freeze resistance of the concrete specimens. The F-T cycles were terminated when the relative dynamic modulus of elasticity was less than 60% or the mass loss was more significant than 5% [30].

As seen from Figure 6a, the dynamic modulus of elasticity of the FB series decreased at a significantly greater rate than that of the FS series with the increase in the number of freeze–thaw cycles. After 200 freeze–thaw cycles, the relative dynamic modulus of elasticity of the FS-1 and FS-2 specimens was 61.6% and 60.2%, respectively. As shown in Figure 6b, the mass loss of the FB-1 and FB-2 specimens after 125 freeze–thaw cycles was 5.4% and 5.1%, respectively. The mass loss of the FS-1 and FS-2 specimens was only 2.6% and 2.4% and, when 200 cycles had passed, the mass loss was 5.7% and 5.2%, respectively, which reached the test’s termination limit. This indicated that the specimens had failed by freezing and thawing. The washed aggregate concretes showed superior freeze–thaw resistance than the unwashed aggregate concretes, with freeze–thaw resistance ratings of F125 for the FB series and F200 for the FS series. The high strength and dense structure of the FS series of concrete increased the pore spacing on the matrix, lowering its initial saturation and increasing the critical value of the freeze–thaw tensile stresses, improving the freeze–thaw resistance of the washed aggregate concretes.

#### 3.3.2. Chloride Ion Penetration Resistance

**Figure 7:**
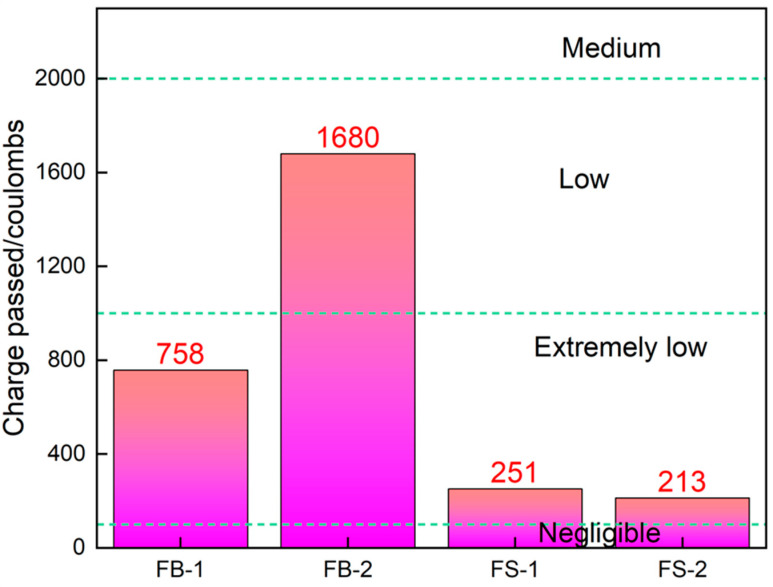
Chloride ion permeability of concrete.

Figure 7 shows the electrical flux diagram of all-solid-waste concrete. Table 6 shows concrete’s chlorine ion permeation resistance grade according to JGJ/T 193-2009. Figure 7 shows that the 28 d electrical fluxes of concrete in FB-1 and FB-2 with unwashed aggregate were 758 C and 1680 C, respectively. The 28 d electrical fluxes of concrete in FS-1 and FS-2 with washed aggregate were 251 C and 213 C, respectively, 66.89% and 87.32% lower than those of FB-1 and FB-2. Combined with Table 3, the chlorine permeation resistance of concrete in the FB-1 group reached the Q-III grade, the chlorine permeation resistance of concrete in the FB-2 group reached the Q-IV grade, and the chlorine permeation resistance of concrete in both the FB-1 and FB-2 groups reached the highest Q-V grade.

Combined with the results of the harmful pore capacity of 50~200 nm in the hardened body of all-solid-waste concrete, it was analyzed that the electrical flux of all-solid-waste concrete had a positive correlation with the harmful pore capacity, i.e., the higher the harmful pore capacity of the slurry, the higher the electrical flux of its concrete and the worse the resistance of the concrete to chlorine ion permeation. In low-strength manufactured-sand concrete, the increase in stone powder content gradually increases the resistance to chloride penetration. Still, in high-strength manufactured-sand concrete, the increase in stone powder content leads to an accelerated decline in resistance to chloride penetration [31]. This is because the chloride solution enters the interior mainly through harmful holes in the concrete, and the smaller the size of the harmful holes and the denser the concrete, the more difficult it is for the chloride ions to enter the interior.

#### 3.3.3. Carbonation

**Figure 8:**
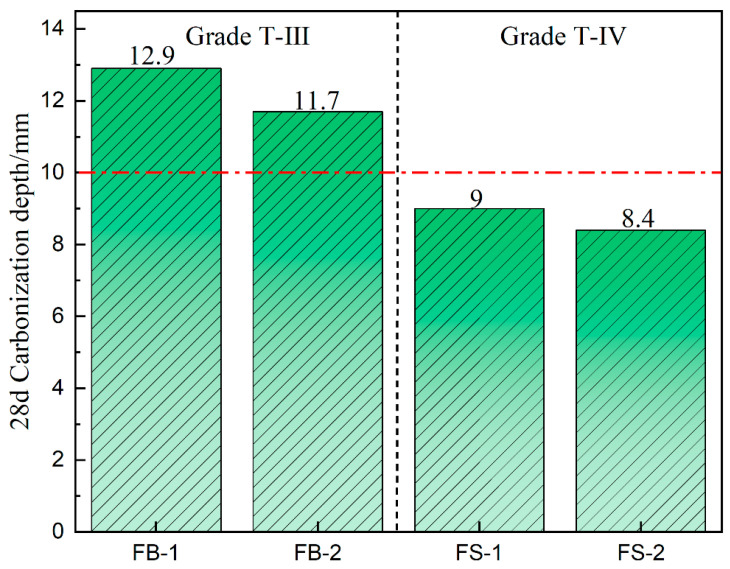
The 28 d carbonation depth of all-solid-waste concrete.

Figure 8 shows the carbonation depth of all-solid-waste concrete carbonated for 28 d. Table 7 shows the carbonation resistance grade of concrete classified in the GB 50164-2011. Figure 8 shows that the carbonation depth of both FB-1 and FB-2 concrete was more than 10 mm and its carbonation resistance was T-III grade. The carbonation depth of FS-1 and FS-2 concretes after aggregate washing was less than 10 mm, the carbonation depth of FS-2 was 8.4 mm, and its carbonation resistance was T-IV grade. Combined with the analysis of the pore structure of the concrete hardened body, the lower the total porosity and the smaller the volume of harmful pores, the higher the degree of compactness of the concrete and the higher the resistance to inward CO_2_ erosion.

#### 3.3.4. Microstructure Characteristics of Concrete

**Figure 9:**
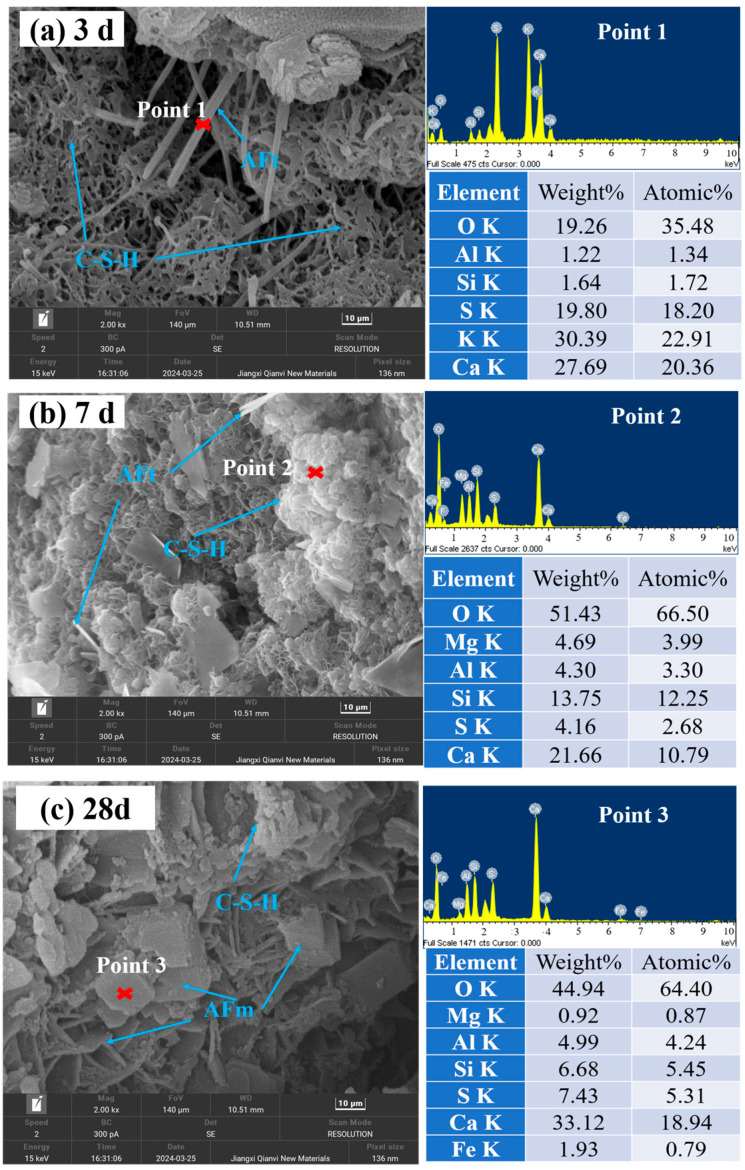
SEM-EDS of slurry specimens.

Figure 9a–c show microscopic images of the slurry specimens hydrated for 3 d, 7 d, and 28 d, respectively. As shown in Figure 9a, the reaction at 3 d of hydration of the slurry specimen produced needle-and-rod AFt and a small amount of agglomerated C-S-H gel [32,33]. AFt was the main contributor to the early strength of the specimen. This showed that few hydration products were generated due to the short maintenance time, the over-lapping was loose, and the whole structure needed to be denser. Figure 9b shows that when the slurry specimen was hydrated for 7 d, much amorphous C-S-H gel and AFt were generated, which overlapped and had a tighter structure than the hydration products at 3 d. Figure 9c shows that a small amount of AFm also appeared in the 28 d hydration products of the concrete specimens with a denser structure [34].

**Figure 10:**
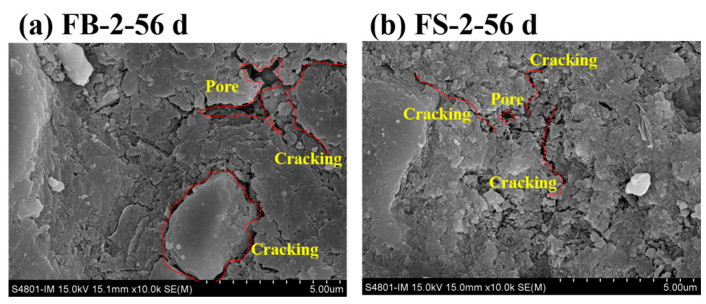
SEM micro-analysis of concrete specimens.

Figure 10a,b show the internal microstructure of concrete specimens of FB-2 and FS-2 that were hydrated for 56 days, respectively. As can be seen, the internal structure of FS-2 was much more structured, with only microcracks appearing. However, the internal cementitious material–aggregate bonding interface of FB-2 was not tight, and the entire internal structure of the sample was loose. Further, the pores were not filled, there were holes internally in addition to microcracks, and the system densification was still low [35]. FB-2 had more cracks in the concrete paste due to the high content of stone powder, which destroyed the filling effect of the cementitious material. The FS-2 system had fewer cracks in the concrete paste, and the overall structure was denser. This was consistent with the results of the strength and durability tests.

### 3.4. Discussions

#### 3.4.1. Effect of Washing Process and Stone Powder

With the washing process, the main change in the quartzite-type waste stone aggregate was the significant reduction of the stone powder and mud lump contents. The contents of stone powder and mud lumps in the unwashed aggregate were 14.6% and 1.88%, respectively, so it qualified as Class III sand. The contents of stone powder and mud lumps in the water-washed aggregate were significantly reduced to 4.5% and 0.19% respectively, reaching the requirements of Class I sand. The content of stone powder in the aggregate, which was converted to the stone powder content in the concrete, was 5% for the FB group and 2% for the FS group. This study demonstrated that high-strength concrete can be prepared from washed quartzite-type tailings waste rock aggregate with solid waste-based cementitious materials.

**Figure 11:**
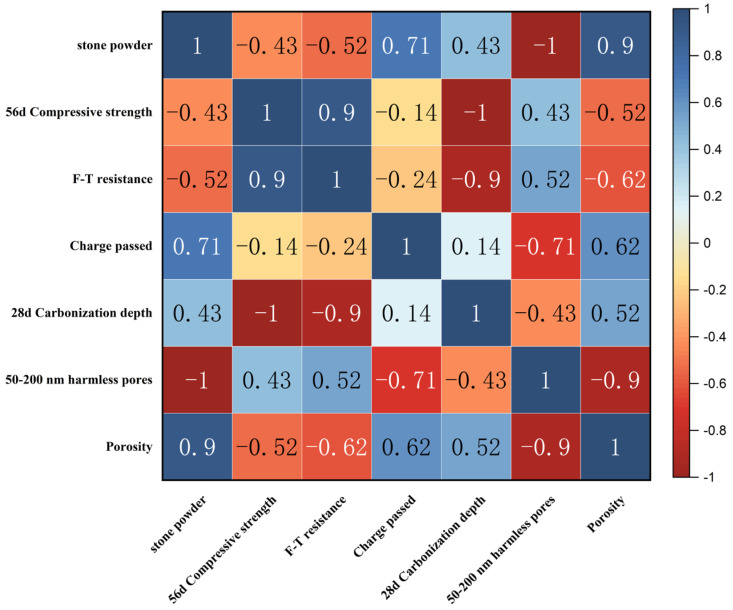
Correlation between all properties of all solid waste-based concrete.

Stone powder is an inert admixture that when activated by mechanical grinding, increases the concrete paste viscosity [36]. However, as the content of stone powder in the aggregate increases, the specific surface area of the aggregate increases and becomes larger, increasing the water demand of concrete significantly [37]. Therefore, the increased stone powder content in the preparation of high-strength concrete needs to be followed by more water to wet the stone powder component, which further affects the mixture densification. The stone powder content showed correlations with compressive strength, frost resistance, electrical flux, carbonation depth, and porosity (Figure 11).

In particular, the stone powder content showed a strong correlation with the porosity of the concrete. The porosity of unwashed aggregate concrete was in the range of 24–27% and the harmful macropores were greater than 50%, while the porosity of washed aggregate was less than 4% and the harmful macropores were less than 40%. Pore size distribution affects the mechanical properties of concrete, so the 28-day compressive strength of the washed aggregate concrete reached 61 MPa, while the 28-day compressive strength of the unwashed aggregate concrete was only 52.7 MPa. In addition, in terms of durability, the mass loss rate and relative dynamic elasticity modulus of the unwashed aggregate concrete, after 50 freezing and thawing cycles, reached the critical value, the resistance to chloride penetration reached the grade of Q-IV, and the carbonation depth was greater than 10 mm. However, after 50 freeze–thaw cycles of washed aggregate concrete, the mass loss rate was less than 1%, the relative dynamic elastic modulus was more than 70%, the resistance to chloride ion permeation reached the highest level of Q-V, and the depth of carbonation was less than 10 mm, so the durability performance was significantly improved.

In comparison with the results of previous studies (Table 8), in this study, all-solid-waste, high-strength concrete was prepared with solid waste-based cementitious materials using iron ore waste rock as an aggregate with excellent mechanical and durability properties.

#### 3.4.2. Environmental and Economic Benefits

From a development perspective, manufactured sand is expected to dominate the market share of construction sand in the future. In this study, iron ore waste rock was used as an aggregate, which, without the need for complex processes and high production standards, has economic and environmental benefits compared to quartz sand and river sand. Compared to cement, solid waste-based cementitious materials are mainly from steel mills and, therefore, have a low price. In China, the price of raw materials is lower than the price of cement. Water washing pretreatment can effectively remove stone powder and mud lumps, and the addition of flocculants to the water can realize recycled water and reduce the impact of high water consumption on the environment [40]. In addition, the 28-day compressive strength of the FS-2 group reached 61 MPa, which satisfies the requirements for high-strength construction materials such as highway and precast components. Therefore, the application of all-solid-waste concrete can not only reduce the harm caused by the accumulation of tailings waste rock to the environment but also has broad application prospects and economic benefits.

## 4. Conclusions

This paper explored the effect of iron ore waste rock on the properties of high-strength concrete through the water-washing process, aiming to better promote the use of waste rock in high-strength concrete. Some main conclusions were drawn:

(1) The washed waste stone aggregate could reduce the stone powder and mud content, which significantly improved the 56-day compressive strength of the concrete from 55.5 MPa to 64.9 MPa. Meanwhile, the porosity was reduced from more than 24% to less than 4%, which significantly improved the denseness of the concrete.
(2) The concrete prepared with washed waste rock aggregate (FS series) was significantly better than the concrete prepared with unwashed aggregate (FB series) in terms of freezing resistance, chloride penetration resistance, and carbonation resistance. The freezing resistance of the FS series concrete reached F200, with chloride penetration resistance of Q-V and carbonation resistance of T-IV, while the freezing resistance of the FB series was only F125, with chloride penetration resistance of Q-IV and carbonation resistance of T-III. The FB series had a frost resistance of F125, a chloride penetration resistance of Q-IV, and a carbonation resistance of T-III.
(3) The hydration products of the washed aggregate concrete were mainly AFt, C-S-H gel, and AFm. The improved performance of the washed aggregate concrete was attributed to the reduced water absorption of aggregates, sufficient hydration reaction, denser internal microstructure, and reduced cracks.

## Figures and Tables

**Table 1:** Chemical composition of cementitious materials (wt.%).

Chemical Composition	CaO	SiO_2_	A1_2_O_3_	MgO	SO_3_	TiO_2_	Fe_2_O_3_	K_2_O	Na_2_O	MnO	Cl
content	50.18	29.10	9.63	5.08	3.15	0.60	1.01	0.45	0.23	0.18	2.01

**Table 2:** Performance indicators of cementitious materials.

Specific Surface Area/(m^2^/kg)	Specific Density/(g/cm^3^)	Water Consumption for Standard Consistency/%	Setting Time/min		Compressive Strength/MPa		Flexural Strength/MPa	
			Initial	Final	3 d	28 d	3 d	28 d
572	2.88	25.65	451	563	30	60.61	1.95	7.89

**Table 3:** Comparison of chemical composition contents of waste rock aggregates (wt.%).

**Chemical Composition**	**SiO_2_**	**Fe_2_O_3_**	**Al_2_O_3_**	**MgO**	**Na_2_O**	**K_2_O**	**CaO**	**SO_3_**	**P_2_O_5_**	**TiO_2_**
Unwashed aggregate	48.74	18.02	17.98	4.76	1.09	3.06	4.26	0.69	0.20	0.48
Washed aggregate	58.62	8.68	19.27	1.75	3.68	2.94	3.78	0.16	0.17	0.60

**Table 4:** Comparison of physical properties between washed and unwashed waste stone aggregates.

	**Mud Content (%)**	**Standardized** **Indicators** **(GB/T 14684)**	**Stone Powder Content (%)**	**MB Value (%)**	**Standardized** **Indicators** **(GB/T 14684)**	**Water Absorption (%)**
Unwashed aggregate	1.88	III	14.6	0.5	III	15.8
Washed aggregate	0.19	I	4.5	0.5	I	5.1

**Table 5:** C60 high-strength concrete mixing proportions (kg/m^3^).

Concrete		Cementitious Materials	Fine Aggregate	Coarse Aggregate	w/b	Water-Reducing Agent
Unwashed	FB-1	550	900	900	154	3.3
	FB-2	600	900	900	168	3.6
Washed	FS-1	550	900	900	154	3.3
	FS-2	600	900	900	168	3.6

**Table 6:** Classification of concrete resistance to chloride permeation (electric flux method).

Grade	Q-I	Q-II	Q-III	Q-IV	Q-V
Charge passed (Coulombs)	≥4000	2000~4000	1000~2000	500~1000	<500

**Table 7:** Classification of concrete carbonation resistance (mm).

Grade	T-I	T-II	T-III	T-IV	T-V
Carbonation depth	≥30	20~30	10~20	0.1~10	<0.1

**Table 8:** Comparison of the results of this study with those of previous studies.

Raw Materials	Stone Powder (%)	28 d Compressive Strength (MPa)	F-T Resistance (RDME%)	Ref.
Waste rock + solid waste-based cementitious materials	4.5	61	66	This study
Portland cement + fly ash + crushed limestone + manufactured sand	5	58.5	72	[38]
Portland cement + fly ash + granulated blast furnace slag powder + silica fume + tuff manufactured sand	5	83.8	―	[39]

## Data Availability

The original contributions presented in this study are included in the article. Further inquiries can be directed to the corresponding authors.
